# Development of Visuospatial Attention in Typically Developing Children

**DOI:** 10.3389/fpsyg.2017.02064

**Published:** 2017-12-06

**Authors:** Gaétan Ickx, Yannick Bleyenheuft, Samar M. Hatem

**Affiliations:** ^1^Institute of Neuroscience, Université catholique de Louvain, Brussels, Belgium; ^2^Physical Medicine and Rehabilitation, Brugmann University Hospital, Brussels, Belgium; ^3^Faculty of Medicine and Faculty of Physical Education and Physiotherapy, Vrije Universiteit Brussel, Brussels, Belgium

**Keywords:** visuospatial attention, children, reference values, development, pointing, line bisection, ogden, star cancellation

## Abstract

The aim of the present study is to investigate the development of visuospatial attention in typically developing children and to propose reference values for children for the following six visuospatial attention tests: star cancellation, Ogden figure, reading test, line bisection, proprioceptive pointing and visuo-proprioceptive pointing. Data of 159 children attending primary or secondary school in the Fédération Wallonie Bruxelles (Belgium) were analyzed. Results showed that the children's performance on star cancellation, Ogden figure and reading test improved until the age of 13 years, whereas their performance on proprioceptive pointing, visuo-proprioceptive pointing and line bisection was stable with increasing age. These results suggest that the execution of different types of visuospatial attention tasks are not following the same developmental trajectories. This dissociation is strengthened by the lack of correlation observed between tests assessing egocentric and allocentric visuospatial attention, except for the star cancellation test (egocentric) and the Ogden figure copy (ego- and allocentric). Reference values are proposed that may be useful to examine children with clinical disorders of visuospatial attention.

## Introduction

Visuospatial attention is the capacity of someone to attend to and to process stimuli in his surrounding space (Posner and Petersen, 1990). In visuospatial attention, different frames of reference can be distinguished: egocentric or allocentric. The egocentric visuospatial representation is important for movement planning and motor control during direct interaction between body and objects, while the allocentric representation is important for determining spatial references in the environment. The interaction between the allocentric and egocentric visuospatial representations allows for spatial processing.

So far, while the development of attention and visuospatial attention has been investigated in infants and children, few of these assessments are really focusing on defining potential deficits in visuospatial attention (i.e., neglect-like) in ego- and allocentric representations. In egocentric neglect (viewer-centered frame of reference), stimuli presented on one side of the person are neglected while, in allocentric neglect (stimuli/objects centered frame of reference), parts of stimuli/objects are neglected regardless of their location to the person (Medina et al., 2009). Assessments currently used to map the development of visuospatial attention or to establish a diagnosis consist in copies of figures (Rey-Osterrieth figure), cancellation tasks (D2 test), attention tests from neuropsychological batteries (e.g., the TEA-Ch and the NEPSY) or tests included in IQ test battery as the block design test of the WISC (Manly et al., 2001; Stinnett et al., 2002; Semrud-Clikeman and Ellison, 2007). Besides the use of neuropsychological tests, visual attention and spatial orienting have been investigated in infants and children by using paradigm cueing visual attention to a spatial location. Analyses of eye pursuit and of saccadic movement in several previous studies have also allowed investigating the development of spatial attention (Johnson et al., 1991, 1994; Colombo, 2001; Rueda et al., 2013). However, these tests could hardly allow identifying specific ego- or allocentric neglect in children with deficits. As it is hypothesized that spatial cognition develops from an egocentric to an allocentric frame of reference (Piaget, 1937; Piaget and Inhelder, 1948), it seems crucial to have assessments testing and documenting the development of both. Furthermore the possibility to follow the development of a deficit from childhood to adulthood requires the use of similar tools along the whole lifespan. Therefore the aim of this study is to investigate the development of visuospatial attention in typically developing children and to create reference values in six assessment tools often used to diagnose visuospatial neglect in adults: star cancellation, Ogden figure, reading test, line bisection, proprioceptive pointing and visuo-proprioceptive pointing. Specifically, differences in the speed of development were expected between tests assessing egocentric spatial attention and tests assessing allocentric spatial attention as the performance of ego- and allocentric visuospatial attention relies on different neural structures and are likely developing in different time windows.

Among the different tests used in this study, three were previously performed in children. Cancellation tasks using assessments similar to those selected for this study have been previously used to investigate visuospatial attention deficits in children (Katz et al., 1998; Laurent-Vannier et al., 2006). Laurent-Vannier showed that the number of teddy bear omissions decreased with age in typically developing children. Letters or digits cancellation tasks highlighted also a relationship between the test performance and the age of the children (Tharpe et al., 2002; Vakil et al., 2009). Line bisection tests were previously used to measure changes in spatial bias in children (Dobler et al., 2001; Failla et al., 2003; Hausmann et al., 2003; Pulsipher et al., 2009). These studies highlighted an effect of age in the response pattern of the line bisection test as well as in the test performance, older children showing smaller deviations. In addition, Hausmann et al. (2003) showed a potential effect of handedness, with a systematic bias towards the side of the hand used in young children and a change to a bias toward the left side, independently of the hand used, in older children. An effect of handedness has also been observed in copying tasks (Braswell and Rosengren, 2002, 2008), potentially affecting the results of the Ogden copy test. Pointing tasks similar to the one used in this study have been used previously (Hay, 1978), showing an age related performance with a non-linear development demonstrating a maximal error at 7 years old.

As handedness could have an effect on the development and results of the different assessments, a secondary aim of this study is to compare visuospatial attention abilities in left and right -handed children in the different ego- and allocentric tests, with a proportion of left and right handed similar to the general population.

Importantly, one of the tests chosen consists in a copy of a drawing. It is well known that drawing abilities are developing during infancy and childhood. Several previous studies illustrate the development of drawing abilities using notably the Draw-a-Person test (Naglieri, 1988) or the Rey-Osterrieth figure copy (Waber and Holmes, 1985; Akshoomoff and Stiles, 1995). These studies highlighted several drawing developments occurring during childhood such as the development of the drawing planning, which showed improvement between 4 and 10 years old with a change in the strategy of the figure copy (Vinter and Marot, 2007; Vinter et al., 2008). Changes were also highlighted in the use of spatial axes during childhood. Nine and eleven-years-old children used spontaneously more often orthogonal and diagonal axes when drawing than 7-years-old children (Lange-Küttner, 2004). Size regulation in drawing, which arises around 5 years, continues to develop during childhood alongside the use of spatial axes. Size regulation development in drawing has been linked to the development of the spatial system and to the use of spatial axes (Lange-Küttner, 1997, 2004, 2008). This development in drawing may influence the performance of children in the copying task. Drawing and copying strategies develop also with age, paralleling the development of writing between 6 and 12 years old which has an influence on the drawing skills (Akshoomoff and Stiles, 1995; Lange-Küttner, 1998; Tabatabaey-Mashadi et al., 2015). Therefore, the development of drawing will be taken into account in our interpretation of the visuo-spatial test based on a copying task.

## Methods

### Participants

One hundred and sixty typically developing (TD) children (82 girls, 78 boys, age range: 4.87–19.1 yrs, 134 right handed) took part in the study. TD children were recruited from seven French-speaking schools in Belgium (Fédération Wallonie Bruxelles). Schools were selected to vary in the average socio-economic status of their school population and adequately represent the average school-aged population in Belgium. The socio-economic status of each school is described by the SEI index (Socio-Economic Index), a synthetic variable computed on the basis of variables as the mean income per inhabitant, mean household income, educational level, activity level, etc. of the area in which the schools are located (see http://www.fapeo.be/wp-content/analyses/analyses_2011/ISEF.pdf for more information). The selected schools had a mean SEI of 12.6 (4, 13, and 20 for the three primary schools and 10, 11, 14, and 16 for the four secondary schools). SEI values ranges between 1 and 20 with higher values representing a higher socio-economic status. The handedness of each child was determined by writing/drawing hand preference (Waldron and Anton, 1995). Children presenting with central nervous system disease (cerebral palsy, ADHD), vestibular disorder, peripheral neurological lesion of the upper extremity, or any motor or sensory impairment of the upper extremity (as appreciated by the parents) were not eligible for this study. This study was approved by the ethics' committee of the Université catholique de Louvain (Belgian ethics file number: B403201316810). Parents (or legal tutors) and children gave their written informed consent after receiving all information regarding the research protocol.

Participants were selected semi-randomly among the children/parents agreeing to participate and confirming that children did not present an excluding clinical condition (as described above). This selection was based on gender and age group (need of minimum 10 children for each age group with a balanced gender ratio). Children were recruited from ages 4 to 19 years old.

### Visuospatial assessments

Children were evaluated at school, individually, in a quiet room, while seated on a chair in front of a table adapted to their height. The duration of assessment (time needed to provide instructions and perform all tests) ranged from 10 min for the eldest (17+ years) to 20 min for the youngest children (5–6 years). Six assessments were performed in a fixed order: Star cancellation, Ogden figure copy, Reading test, Line bisection, Proprioceptive pointing and visuo-proprioceptive pointing. The children used their dominant hand to perform the different assessments. The instructions for testing can be found in Supplementary Table 1.

#### Star cancellation

The star cancellation test consists of a page covered with 108 stars (52 big and 56 small) and with distractors (words and letters). The middle of the page is aligned with the subject's midline and the subject is asked to cross out all the small stars. The subject is instructed to put his pencil down as soon as he thinks all small stars are crossed out. Primary variables are the total number of omissions and the time needed to complete the task. For scoring, the page is divided in 4 columns, 1 left (16 small stars), 1 right (16 small stars), 1 center-left (14 small stars), and 1 center-right (10 small stars). The total number of left omissions is the sum of the left column and center-left column omitted stars. The total number of right omissions is the sum of right column and center-right column omitted stars (Wilson et al., 1987). Star cancellation assesses egocentric visuospatial attention as the stars are considered being either on the right or on the left relative to the child (Keller et al., 2005).

#### Ogden figure copy

Ogden figure copy is a drawing test in which the subject is instructed to copy a drawing. The drawing includes four trees, two on the left and two on the right side of a house located in the middle of the page; the house has a door and four windows (two on the left and two on the right side of the house). The interest of this test is to detect omissions of specific elements in the copy, and not to evaluate the quality of the drawing nor the strategy used by the child during the copy. The test is scored on a scale from 0 to 4, where 0 is a copy without omissions, 1 a copy with omission of a right or left window, 2 a copy with omission of the left or right part of the house or of a tree, 3 a copy with omission of a complete tree and 4 a copy with omission of a complete tree plus another left or right part of the figure. The time needed to complete the task is recorded (Ogden, 1985). Ogden figure copy assesses both ego- and allocentric visuospatial attention (Medina et al., 2009). Omissions of parts of the drawing on the left side or on the right side of the sheet (left or right side relative to the child) are considered as egocentric errors (viewer-based neglect), while omissions of left or right side of the trees or of the house (independently of their position relative to the child) are considered as allocentric errors (stimuli-based neglect). For example, the copy of the house and of only the trees located on the right would be considered an egocentric error, while the copy of the house and of the right part of each tree (trees located on the right and on the left of the house) would be an allocentric error.

#### Reading test

For the reading test, the subject is instructed to read a text out loud. First-graders and children younger than 7 years old were excluded from this assessment as they lack sufficient reading skills. The text is presented on an A4 sheet of paper in landscape position. The text, written in lower case is composed of 9 lines of text for a total of 77 words. A reproduction of the text can be found in Supplementary Materials. Scoring includes the number of word omissions on either lateral sides of the text, the time taken to read the text, and the number of substitutions on either sides of the text (Reinhart et al., 2013). Both ego- and allocentric errors can be detected by this test (Medina et al., 2009). Omissions of the left or right part of the text are egocentric (viewer-based) errors, while omissions of the left or right part of words independently of their position relative to the child are allocentric (stimuli-based) errors. For example: “longtemps” read as “temps” is an omission of the left part of the word and still an existing word in French, or read as “long” which is an omission of the right part of the word but still an existing word in French.

#### Line bisection

The line bisection test consists of three pages, each containing ten lines of different lengths to bisect (pages 1: 3 lines of 5 cm, 3 lines of 15 cm, 2 lines of 20 cm and 2 lines of 10 cm; page 2: 5 lines of 13.4 cm, 3 lines of 9.4 cm and 2 lines of 4.7 cm; page 3: 3 lines of 2.6 cm, 2 lines of 7.9 cm, 2 lines of 10.5 cm). The subject is instructed to mark the exact middle of each line with a pencil. The percentage error from the line bisection is calculated with the following formula:

$$\begin{array}{l} \text{Error}=\left(\text{b}-\text{a}\right)/{\text{a}}^{*}100 \end{array}$$

Where a = half of the line length, b the distance between the beginning of the line and the mark made by the child. An error toward the left side of space is recorded as a negative value (Scarisbrick et al., 1987). Line bisection test assesses allocentric visuospatial attention as children will present a deviation relative to the center of the line.

#### Proprioceptive pointing

For proprioceptive pointing the subject is seated in front of a table, his body midline aligned with the center of a paper sheet taped on the table. The sheet of paper is covered with radiating lines indicating the error in degrees from the center of the sheet. Subjects are blindfolded and asked to point toward their perceived body midline on the paper by moving their index finger forward. Each subject performs four pointings. The average value of the four pointings is calculated as the average pointing error. A error toward the left side of space is recorded as a negative value (Riquelme et al., 2015). Proprioceptive pointing assesses egocentric visuospatial attention as the deviation of the pointing will be relative to the body midline of the children.

#### Visuo-proprioceptive pointing

As described in Riquelme et al. (2015), for visuo-proprioceptive pointing the children are seated in front of a half-open wooden box, closed on one side by a transparent Plexiglas indicating the degrees of error from the center. The base of the box is an isosceles right-angled triangle, with an opening on the hypotenuse side (see Frassinetti et al., 2002 for a complete description of the box). The body midline of the subjects is aligned with the 0° axis of the box (middle of the box). In this position, children are asked to point inside the box (without visual feedback of the range of motion of the arm) towards a target appearing at three different positions above the box. The three different target positions are at 0°, +21° (right side of space) and −21° (left side of space). Each target is presented three times. The mean visuo-proprioceptive pointing error is calculated for each target. The average visuo-proprioceptive pointing error is calculated as the average of all 9 pointings. An error toward the left side of space is recorded as a negative value. Visuo-proprioceptive pointing assesses allocentric visuospatial attention as the deviation of each pointing will be relative to the targets independently of their position.

### Statistical analyses

IBM SPSS 22 package was used for statistical analysis. The significance level was set at *p* < 0.05. Descriptive statistics were computed. The normality of distribution and the homogeneity of variances were assessed using Kolmogorov-Smirnov test (normality) and the Levene's test (homogeneity of variance) for each variable in each age group. Tests used to assess handedness, gender and age effects are provided at the start of each result description.

## Results

### Sample description

From the sample recruited at the start of the study, one child (boy, 4.94 years old, left handed) was discarded of the study because he did not understand instructions and could not complete the assessments. Another child (boy, 4.88 years old, left handed) was unable to perform the visuo-proprioceptive pointing task and therefore a score for this task was lacking for this child. The final sample included 159 TD children (82 girls, 77 boys, age range: 4.87–19.1 yrs, 134 right handed). Children younger than five years old were included in the age group of 5 years (*n* = 3, age = 4.88 ± 0.015). Children older than 17 (*n* = 2, age = 18.56 ± 0.761) were included in the 17+ age group. Table 1 is presenting the percentage of left and right handed children per age group.

**Table 1:** Description of the demographic characteristics and sample size in each age group.

**Age group**	**Mean age (*SD*)**	**% left handed (*N*)**	**% right handed (*N*)**	***N***
5	5y2m (3m21d)	22% (2)	78% (7)	9
6	6y5m (3m21d)	27% (3)	73% (8)	11
7	7y6m (3m18d)	14% (2)	86% (12)	14
8	8y6m (3m24d)	8% (1)	92% (11)	12
9	9y7m (2m21d)	7% (1)	93% (13)	14
10	10y5m (3m15d)	15% (2)	85% (11)	13
11	11y4m (4m)	25% (2)	75% (6)	8
12	12y6m (3m18d)	23% (3)	77% (10)	13
13	13y5m (3m3d)	24% (4)	76% (13)	17
14	14y4m (3m)	8% (1)	92% (12)	13
15	15y4m (3m9d)	8% (1)	92% (11)	12
16	16y6m (4m6d)	8% (1)	92% (11)	12
17+	17y7m (6m27d)	18% (2)	82% (9)	11

In case of a normal data distribution, outliers >2.5 SD (i.e., SD of this age group) were discarded (considered as an incidental measurement error) and data were expressed as mean and standard error. In case of a non-Gaussian distribution, outliers > 97th percentile were discarded and data expressed as median and percentiles. Following this procedure, solely two subjects tested were considered as outliers for the visuo-proprioceptive pointing and discarded (values of visuo-proprioceptive mean error of +4.88° and +4.77°, respectively at 7 and 9 years old).

The age-related variance of the socio-economic status (as measured by the school SEI) was investigated, using a Kruskal-Wallis test with the factor AGE as between-subjects factor (13 age groups from 5 years to 17+ years). SEI did not differ between the different age groups. Because of the equal distribution of SEI between the different age groups, SEI was not used as a covariate in further analyses [K-W (factor: age-groups): χ2_(12, N = 159)_ = 10.24; *p* = 0.595].

### Visuospatial assessments

One way analyses of variance (ANOVA) with between-subjects factors GENDER (male vs. female) were used for parametric variables to test a potential gender effect. For non-parametric variables, a Kruskal-Wallis test was performed. Gender did not interact significantly with any variable. Therefore the following analyses were performed on the whole sample without splitting boys and girls.

The Age effect was investigated between 13 age groups for all variables. Relative values were created for each assessment and each age group as the 95% confidence interval (mean ± 2 SD) for variables with a Gaussian distribution, and as the 95th percentile for variables with a non-Gaussian distribution.

**Figure 1:**
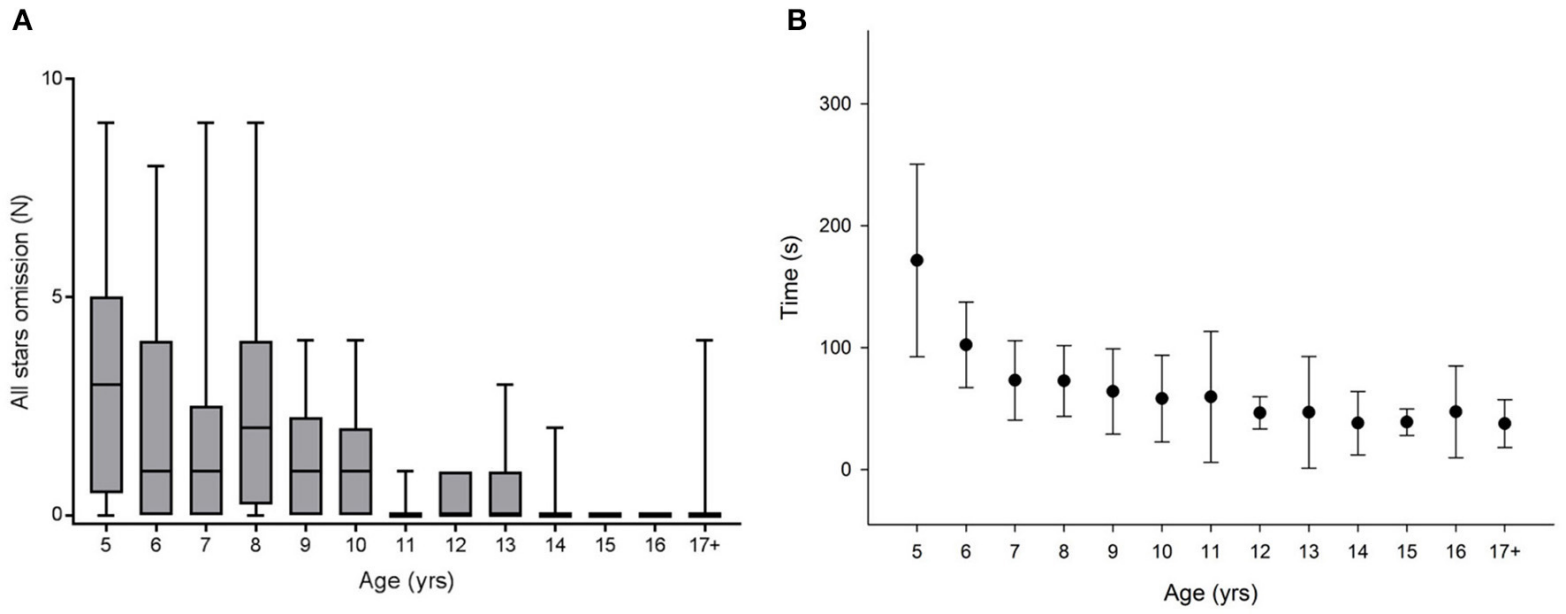
Significant differences between age groups were observed for **(A)** total star omission [Kruskal-Wallis Chi square: χ2_(12, N = 159)_ = 56.693; *p* < 0.001; *post hoc* pairwise comparison: 8 years vs. 14–17+ years: *p* < 0.042) as well as for **(B)** the time taken to complete the test [Kruskal-Wallis Chi square: χ2_(12, N = 159)_ = 97.271; *p* < 0.001; *post hoc* pairwise comparison: 5 years vs. 12–17+: *p* < 0.002].

**Figure 2:**
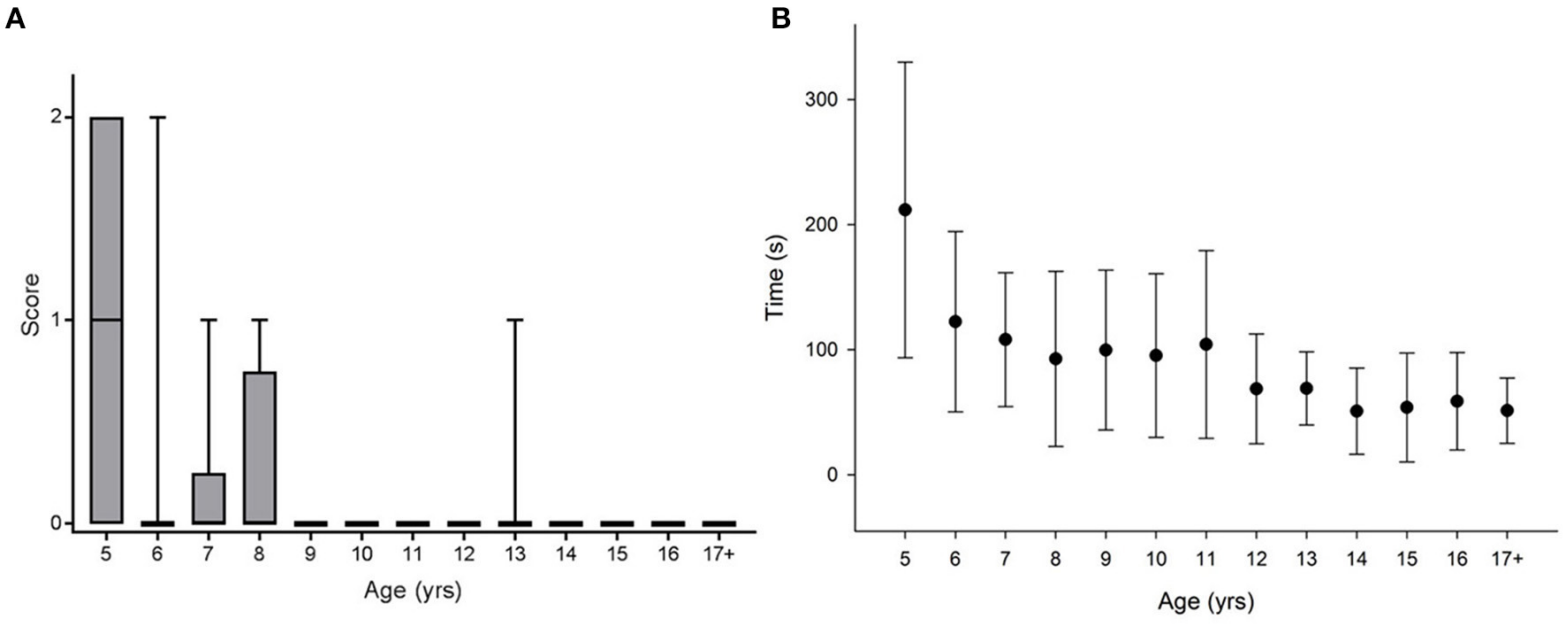
Significant differences between age groups were observed for **(A)** the Ogden figure score [Kruskal-Wallis Chi square: χ2_(12, N = 159)_ = 52.496; *p* < 0.001; *post hoc* pairwise comparison: 5 years vs. 6–17+ years: *p* < 0.013) as well as for **(B)** the time taken to complete the test [Kruskal-Wallis Chi square: χ2_(12, N = 159)_ = 96.573; *p* < 0.001; *post hoc* pairwise comparison: 5 years vs. 12–17+: *p* < 0.001].

**Figure 3:**
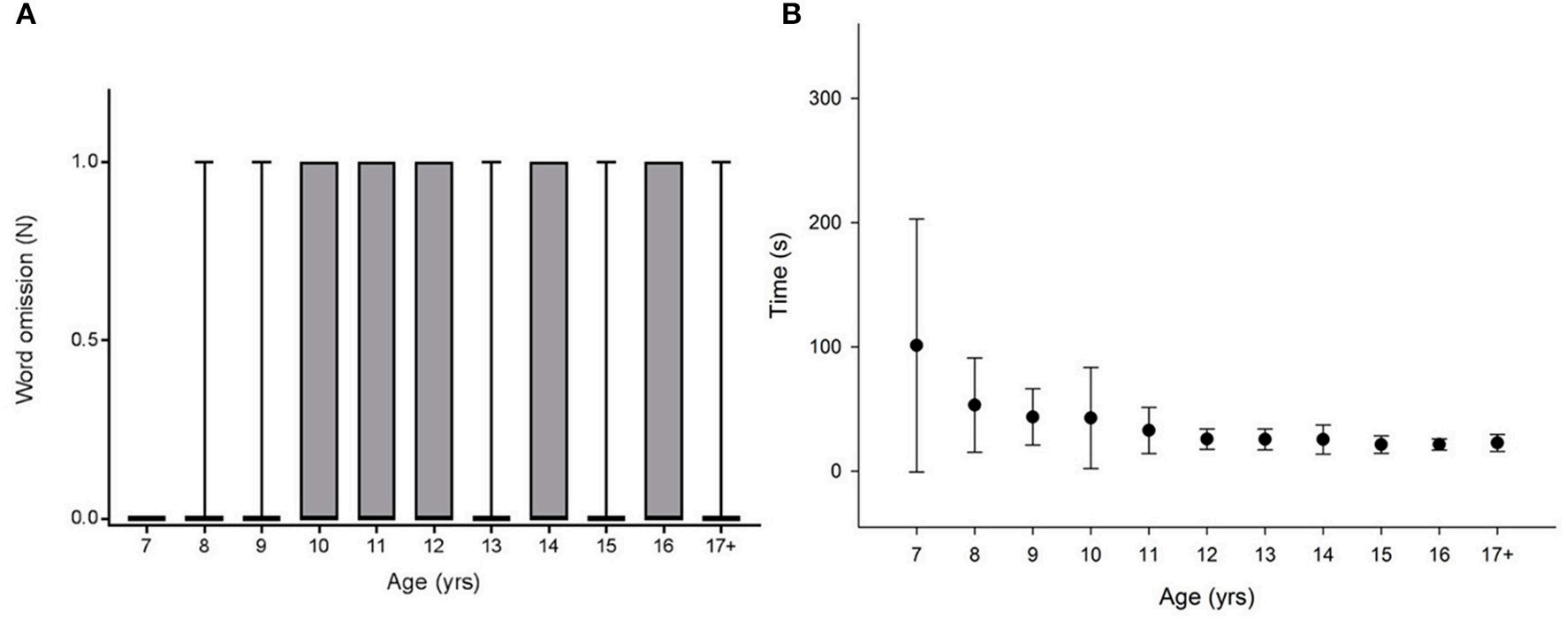
No age related difference was found for **(A)** the number of omitted word [Kruskal Wallis: χ2_(10, N = 134)_ = 10.072; *p* = 0.434]. A significant difference between age groups was observed for **(B)** the reading time [Kruskal-Wallis Chi square: χ2_(10, N = 134)_ = 95.065; *p* < 0.001; *post hoc* pairwise comparison: 7 years vs. 12–17+ years: *p* < 0.001).

**Figure 4:**
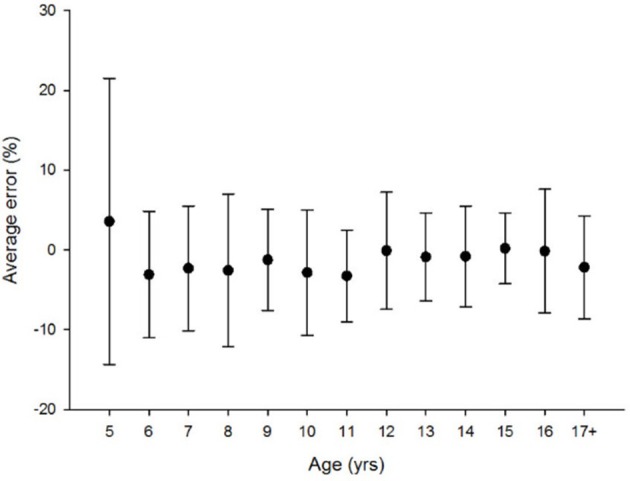
No age related difference was found [Kruskal-Wallis Chi square: χ2_(12, N = 159)_ = 17.566; *p* = 0.13].

**Figure 5:**
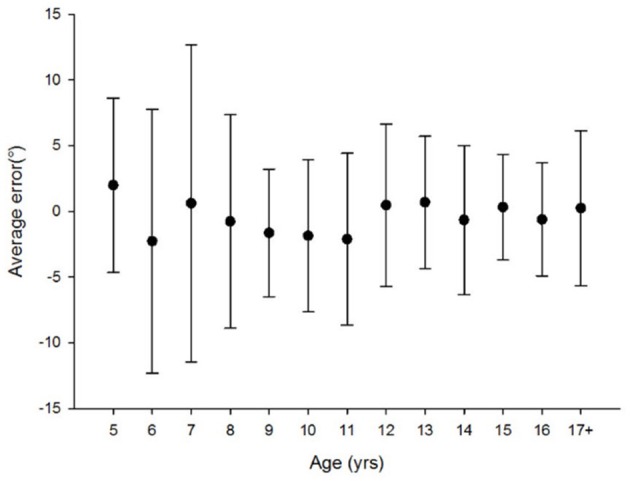
No age related difference was found [Kruskal-Wallis Chi square: χ2_(12, N = 159)_ = 18.866; *p* = 0.09].

**Figure 6:**
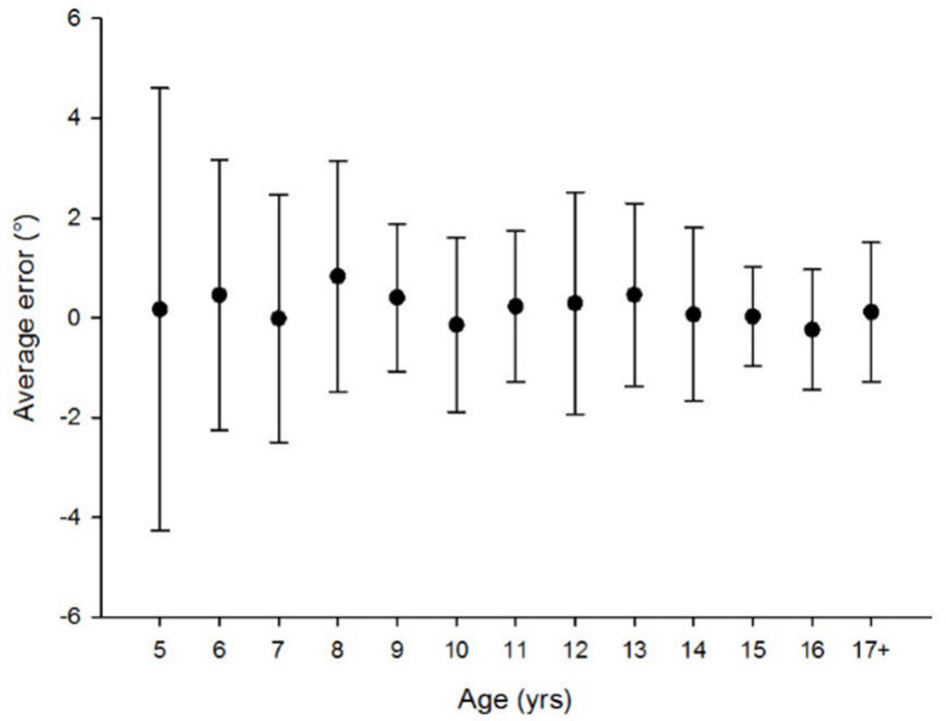
No age related difference was found [Kruskal-Wallis Chi square: χ2_(12, N = 156)_ = 14.749; *p* = 0.255].

Figures 1A, 2A, 3A, 4–**6** show the data distribution per age for each visuospatial assessment. Figures 1B, 2B, 3B show, respectively, the distribution of the variable ‘time’ for the star cancellation test, Ogden figure copy test and the reading test. The reference values of each visuospatial attention test per age group are described in Table 2 for variables with a Gaussian distribution and in Table 3 for variables with a non-Gaussian distribution. Supplementary Table 2 shows the conversion between raw-score and z-score (variable with a Gaussian distribution) or percentile (variables with a non-Gaussian distribution).

**Table 2:** Pediatric reference values by age group for the variables with a Gaussian distribution.

**Test**	**Variable**	**Age group (years)**	**Subjects (*n* =)**	**Mean**	**Standard deviation**	**95%-confidence interval**
Line bisection	Average error (%)	5	9	3.58	8.964	−14.35 to 21.51
		6	11	−3.08	3.970	−11.02 to 4.86
		7	14	−2.30	3.905	−10.11 to 5.51
		8	12	−2.56	4.784	−12.13 to 7.01
		9	14	−1.24	3.164	−7.56 to 5.09
		10	13	−2.83	3.934	−10.7 to 5.04
		11	8	−3.26	2.877	−9.01 to 2.5
		12	13	−0.10	3.672	−7.44 to 7.25
		13	17	−0.89	2.738	−6.36 to 4.59
		14	13	−0.81	3.155	−7.12 to 5.5
		15	12	0.20	2.223	−4.25 to 4.64
		16	12	−0.16	3.878	−7.91 to 7.6
		17+	11	−2.19	3.221	−8.63 to 4.26
Proprioceptive pointing	Average error (°)	5	9	1.97	3.315	−4.66 to 8.6
		6	11	−2.27	5.011	−12.29 to 7.75
		7	14	0.61	6.030	−11.45 to 12.67
		8	12	−0.77	4.060	−8.89 to 7.35
		9	14	−1.64	2.425	−6.49 to 3.21
		10	13	−1.87	2.899	−7.66 to 3.93
		11	8	−2.13	3.265	−8.66 to 4.41
		12	13	0.46	3.087	−5.71 to 6.64
		13	17	0.68	2.517	−4.36 to 5.71
		14	13	−0.67	2.829	−6.33 to 4.99
		15	12	0.31	2.012	−3.71 to 4.34
		16	12	−0.63	2.149	−4.92 to 3.67
		17+	11	0.23	2.951	−5.67 to 6.13
Visuo-proprioceptive pointing	Average error (°)	5	8	0.17	2.217	−4.27 to 4.6
		6	11	0.45	1.354	−2.25 to 3.16
		7	13	−0.02	1.240	−2.5 to 2.46
		8	12	0.83	1.161	−1.49 to 3.15
		9	13	0.40	0.738	−1.07 to 1.88
		10	13	−0.15	0.873	−1.89 to 1.6
		11	8	0.22	0.754	−1.28 to 1.73
		12	13	0.29	1.111	−1.93 to 2.51
		13	17	0.46	0.916	−1.37 to 2.29
		14	13	0.06	0.870	−1.67 to 1.8
		15	12	0.02	0.494	−0.97 to 1.01
		16	12	−0.24	0.601	−1.44 to 0.96
		17+	11	0.11	0.696	−1.28 to 1.5
	Right target error (°)	5	8	−1.67	1.886	−5.44 to 2.1
		6	11	−0.70	1.912	−4.52 to 3.13
		7	13	−1.29	1.844	−4.97 to 2.4
		8	12	−0.46	2.500	−5.46 to 4.54
		9	13	−0.43	1.165	−2.76 to 1.9
		10	13	−1.69	1.023	−3.74 to 0.35
		11	8	−1.08	1.707	−4.5 to 2.33
		12	13	0.26	1.973	−3.69 to 4.2
		13	17	0.24	1.928	−3.62 to 4.09
		14	13	−0.56	1.595	−3.75 to 2.63
		15	12	−0.51	0.601	−1.72 to 0.69
		16	12	−0.47	1.403	−3.28 to 2.33
		17+	11	0.09	1.156	−2.22 to 2.4
	Central target error (°)	5	8	0.42	2.158	−3.9 to 4.73
		6	11	1.03	1.760	−2.49 to 4.55
		7	13	0.62	2.132	−3.64 to 4.88
		8	12	0.58	0.889	−1.19 to 2.36
		9	13	0.93	1.675	−2.42 to 4.28
		10	13	−0.26	0.992	−2.24 to 1.73
		11	8	0.17	1.024	−1.88 to 2.21
		12	13	0.10	1.125	−2.15 to 2.35
		13	17	0.51	1.259	−2.01 to 3.03
		14	13	0.05	0.792	−1.53 to 1.63
		15	12	−0.11	0.845	−1.8 to 1.58
		16	12	−0.44	0.499	−1.44 to 0.55
		17+	11	−0.24	0.858	−1.96 to 1.47
	Left target error (°)	5	8	1.75	3.156	−4.56 to 8.06
		6	11	1.03	1.906	−2.78 to 4.84
		7	13	1.67	2.816	−3.97 to 7.3
		8	12	2.36	1.494	−0.63 to 5.35
		9	13	1.64	2.073	−2.5 to 5.79
		10	13	1.51	1.751	−1.99 to 5.02
		11	8	1.58	1.571	−1.56 to 4.73
		12	13	0.51	1.778	−3.04 to 4.07
		13	17	0.63	1.092	−1.56 to 2.81
		14	13	0.71	1.313	−1.92 to 3.33
		15	12	0.69	1.176	−1.66 to 3.05
		16	12	0.19	0.834	−1.47 to 1.86
		17+	11	0.48	0.970	−1.46 to 2.43

**Table 3:** Pediatric reference values by age group, for the variables with a non-Gaussian distribution.

**Test**	**Variable**	**Age group (yrs)**	**Subjects (*n* =)**	**Median**	**Interquartile range**	**25th−95th Percentile**
Star cancellation	All stars omission (*n* =)	5	9	3.00	4.50	0.5–9
		6	11	1.00	4.00	0–8
		7	14	1.00	2.50	0–9
		8	12	2.00	3.75	0.25–9
		9	14	1.00	2.25	0–4
		10	13	1.00	2.00	0–4
		11	8	0.00	0.00	0–1
		12	13	0.00	1.00	0–1
		13	17	0.00	1.00	0–3
		14	13	0.00	0.00	0–2
		15	12	0.00	0.00	0–0
		16	12	0.00	0.00	0–0
		17+	11	0.00	0.00	0–4
	Left stars omission (*n* =)	5	9	0.00	3.50	0–9
		6	11	1.00	3.00	0–4
		7	14	1.00	2.25	0–5
		8	12	1.00	2.00	0–4
		9	14	0.00	0.25	0–2
		10	13	0.00	1.50	0–4
		11	8	0.00	0.00	0–1
		12	13	0.00	0.00	0–0
		13	17	0.00	0.50	0–3
		14	13	0.00	0.00	0–0
		15	12	0.00	0.00	0–0
		16	12	0.00	0.00	0–0
		17+	11	0.00	0.00	0–4
	Right stars omission (*n* =)	5	9	1.00	1.50	0–4
		6	11	0.00	1.00	0–4
		7	14	0.00	0.00	0–4
		8	12	1.00	2.75	0–5
		9	14	0.50	1.25	0–4
		10	13	0.00	1.00	0–2
		11	8	0.00	0.00	0–0
		12	13	0.00	1.00	0–1
		13	17	0.00	0.00	0–1
		14	13	0.00	0.00	0–2
		15	12	0.00	0.00	0–0
		16	12	0.00	0.00	0–0
		17+	11	0.00	0.00	0–0
	Time (s)	5	9	174.00	72.00	135–222
		6	11	100.00	15.50	93.5–140
		7	14	74.00	16.75	63.25–113
		8	12	75.00	28.00	57.5–91
		9	14	64.50	30.00	48–89
		10	13	55.00	25.00	44–103
		11	8	44.50	42.00	44–111
		12	13	47.00	8.00	42.5–57
		13	17	40.00	12.50	35–125
		14	13	39.00	11.50	32.5–61
		15	12	38.50	8.25	36–46
		16	12	42.00	13.25	36.25–91
		17+	11	34.00	8.00	32–58
Ogden figure copy	Score	5	9	1.00	2.00	0–2
		6	11	0.00	0.00	0–2
		7	14	0.00	0.25	0–1
		8	12	0.00	0.75	0–1
		9	14	0.00	0.00	0–0
		10	13	0.00	0.00	0–0
		11	8	0.00	0.00	0–0
		12	13	0.00	0.00	0–0
		13	17	0.00	0.00	0–1
		14	13	0.00	0.00	0–0
		15	12	0.00	0.00	0–0
		16	12	0.00	0.00	0–0
		17+	11	0.00	0.00	0–0
	Time (s)	5	9	212.00	96.50	164–309
		6	11	128.00	47.00	97–173
		7	14	103.00	33.50	93.5–167
		8	12	82.50	36.75	73.25–175
		9	14	93.00	32.75	75–180
		10	13	79.00	42.00	72–176
		11	8	93.00	58.00	72–173
		12	13	65.00	26.00	50.5–130
		13	17	68.00	17.00	60–102
		14	13	52.00	19.00	40–94
		15	12	53.50	30.00	37.25–95
		16	12	60.00	26.75	43.25–101
		17+	11	60.00	25.00	38–65
Reading	Word omission (*n* =)	7	14	0.00	0.00	0–0
		8	12	0.00	0.00	0–1
		9	14	0.00	0.00	0–1
		10	13	0.00	1.00	0–1
		11	8	0.00	1.00	0–1
		12	13	0.00	1.00	0–1
		13	17	0.00	0.00	0–1
		14	13	0.00	1.00	0–1
		15	12	0.00	0.00	0–1
		16	12	0.00	1.00	0–1
		17+	11	0.00	0.00	0–1
	Word substitution (*n* =)	7	14	2.00	2.50	0–4
		8	12	0.00	1.00	0–2
		9	14	0.00	0.00	0–1
		10	13	0.00	1.00	0–2
		11	8	0.00	0.00	0–1
		12	13	0.00	0.00	0–1
		13	17	0.00	0.00	0–0
		14	13	0.00	0.00	0–1
		15	12	0.00	0.00	0–1
		16	12	0.00	0.00	0–0
		17+	11	0.00	0.00	0–1
	Time (s)	7	14	85.50	94.00	58–195
		8	12	50.50	29.25	35.5–93
		9	14	43.00	13.75	37.5–68
		10	13	38.00	15.50	31–106
		11	8	31.50	15.25	31–49
		12	13	26.00	4.50	24.5–32
		13	17	25.00	5.50	22–34
		14	13	23.00	8.00	21.5–38
		15	12	21.00	3.50	19.25–30
		16	12	21.50	3.25	20.25–25
		17+	11	23.00	5.00	20–29

#### Star cancellation

##### Star omission

As the distributions were not Gaussian, handedness was first investigated using a Kruskal-Wallis. An effect of handedness was found for the number of left omitted stars [K-W (factor: right- vs. left-handed): χ2_(1, N = 159)_ = 6.569; *p* = 0.01]. Left handed children omitted more stars on the left side (number of omitted stars on the left side: right handed: 0.44 ± 0.954; left-handed:1.48 ± 2.275).

An ANOVA on ranks was also performed to investigate age effects. Significant differences between age groups were observed for total star omission, younger children omitting more stars than older children [Figure 1A, Kruskal-Wallis Chi square: χ2_(12, N = 159)_ = 56.693; *p* < 0.001; *post hoc* pairwise comparison: 8 years vs. 14–17+ years: *p* < 0.042) as well as for right and left omissions [Kruskal-Wallis Chi square: χ2_(12, N = 159)_ = 39.483; *p* < 0.001 and Kruskal-Wallis Chi square: χ2_(12, N = 159)_ = 50.736; *p* < 0.001]. In separate analyses made in left handed or right handed children, no age related difference was detected in left handed children for total star omission [Kruskal-Wallis Chi square: χ2_(12, N = 25)_ = 18.385; *p* = 0.104] nor for right and left omission [Kruskal-Wallis Chi square: χ2_(12, N = 25)_ = 17.308; *p* = 0.138 and Kruskal-Wallis Chi square: χ2_(12, N = 25)_ = 16.743; *p* = 0.16]. However, the sample size of left-handed children (*n* = 25) was small with on average only two left-handed children per age group. In right handed children, age related differences were highlighted in for total star omission [Kruskal-Wallis Chi square: χ2_(12, N = 134)_ = 47.294; *p* < 0.001] and for right and left omission [Kruskal-Wallis Chi square: χ2_(12, N = 134)_ = 36.779; *p* < 0.001 and Kruskal-Wallis Chi square: χ2_(12, N = 134)_ = 39.439; *p* < 0.001].

##### Time

Due to a Gaussian distribution and a homogeneity of the variances (Levene's test), handedness was tested using an ANOVA. No effect of handedness was found [ANOVA (factor: right- vs. left-handed): *F*_(1, 158)_ = 0.670, *p* = 0.414].

As a consequence of the difference in the homoscedasticity (Levene's test), an ANOVA on ranks was used to test the effect of age. Significant differences between age groups were observed [Figure 1B, Kruskal-Wallis Chi square: χ2_(12, N = 159)_ = 97.271; *p* < 0.001; *post hoc* pairwise comparison: 5 years vs. 12–17+: *p* = 0.002).

#### Ogden figure copy

##### Score

As the distribution was not Gaussian, handedness was first investigated using a Kruskal-Wallis. No effect of handedness was found [K-W (factor: right- vs. left-handed): χ2_(1, N = 159)_ = 0.54; *p* = 0.815]. A Kruskal-Wallis was also performed to investigate age effects. Significant differences between age groups were observed [Figure 2A, Kruskal-Wallis Chi square: χ2_(12, N = 159)_ = 52.496; *p* < 0.001; *post hoc* pairwise comparison: 5 years vs. 6–17+ years: *p* = *0.0*13].

##### Time

The Gaussian distribution and the homogeneity of the variances (Levene's test) allowed testing handedness using an ANOVA. No effect of handedness was found [ANOVA (factor: right- vs. left-handed): *F*_(1, 158)_ = 0.108, *p* = 0.743]. As a consequence of the difference in the homoscedasticity (Levene's test), an ANOVA on ranks was used to test the effect of age. Significant differences between age groups were observed [Figure 2B; Kruskal-Wallis Chi square: χ2_(12, N = 159)_ = 96.573; *p* < 0.001; *post hoc* pairwise comparison: 5 years vs. 12–17+: *p* < 0.001].

#### Reading test

##### Word omission

As the distribution was not Gaussian, handedness was first investigated using a Kruskal-Wallis. An effect of handedness was found [K-W (factor: right- vs. left-handed): χ2_(1, N = 134)_ = 4.916; *p* = 0.027]. A Kruskal-Wallis was also performed to investigate age effects. No age-related difference was observed [Figure 3A, K-W: χ2_(10, N = 134)_ = 10.072; *p* = 0.434] in the whole sample nor in left handed children [χ2_(10, N = 19)_ = 13.142; *p* = 0.216] or in right handed children [χ2_(10, N = 115)_ = 7.501; *p* = 0.677].

##### Word substitution

As the distribution was not Gaussian, handedness was first investigated using a Kruskal-Wallis. No effect of handedness was found [K-W (factor: right- vs. left-handed): χ2_(1, N = 134)_ = 2.301; *p* = 0.129]. A Kruskal-Wallis was also performed to investigate age effects. An age-related difference was observed [χ2_(10, N = 134)_ = 28.692; *p* = 0.001; *post hoc* pairwise comparison: 7 years vs. 12–17+ years: *p* < 0.011].

##### Reading time

The Gaussian distribution and the homogeneity of the variances (Levene's test) allowed testing handedness using an ANOVA. No effect of handedness was found [ANOVA (factor: right- vs. left-handed): *F*_(1, 133)_ = 0.436, *p* = 0.510]. As a consequence of the difference in the homoscedasticity (Levene's test), an ANOVA on ranks was used to test the effect of age. An ANOVA on ranks was performed to investigate age effects. Significant differences between age groups were observed [Figure 3B: Kruskal-Wallis Chi square: χ2_(10, N = 134)_ = 95.065; *p* < 0.001; *post hoc* pairwise comparison: 7 years vs. 12–17+ years: *p* < 0.001].

#### Line bisection

The Gaussian distribution and the homogeneity of the variances (Levene's test) allowed testing handedness using an ANOVA. An effect of handedness was found [ANOVA (factor: right- vs. left-handed): *F*_(1, 158)_ = 13.994, *p* < 0.001]. Left-handed children bisected significantly more towards the left side of space than right-handed children (right-handed: −0.71% ± 4.117; left-handed: −3.99% ± 3.452). Homoscedasticity between age groups was investigated using Levene's test and a difference was found [*F*_(12;146)_ = 3.134; *p* = 0.001]. Therefore, Kruskal-Wallis was performed to investigate age effects. There were no age-related differences for line bisection [Figure 4: Kruskal-Wallis Chi square: χ2_(12, N = 159)_ = 17.566; *p* = 0.13]. Analyses performed separately in left handed and right handed children, demonstrated no age-related differences for this variable [left handed children: Kruskal-Wallis Chi square: χ2_(12, N = 25)_ = 11.145; *p* = 0.517; right handed children: Kruskal-Wallis Chi square: χ2_(12, N = 134)_ = 16.689; *p* = 0.162]. The mean bisection bias of the overall sample was significantly different from zero [*t*_(158)_ = −3.698, *p* < 0.001] indicating that children bisect significantly away from the midline of peripersonal space toward the left side, the same result was found in left handed and right handed children [left handed: *t*_(24)_ = −5.78, *p* < 0.001; right handed: *t*_(133)_ = −2.001, *p* = 0.047].

##### Complementary investigation: the effect of line length

Effect of line length on bisection error was investigated using One Ways repeated measures ANOVA with the factor line length (4 levels: lines of 5 cm or less, between 5 and 10 cm, between 10 and 15 cm and line of more than 15 cm) as within subject factor. Analyses showed an overall difference of bisection error between line lengths (*F* = 7.811, *p* < 0.001). Relative values for the different lines lengths are reported in Supplementary Table 3.

#### Proprioceptive pointing

The Gaussian distribution and the homogeneity of the variances (Levene's test) allowed testing handedness using an ANOVA. No effect of handedness was found [ANOVA (factor: right- vs. left-handed): *F*_(1, 158)_ = 0.368, *p* = 0.545]. Difference of variance between age groups was investigated using Levene's test and a difference was found [*F*_(12;146)_ = 2.526; *p* = 0.005]. Therefore, a Kruskal-Wallis was performed to investigate age effects. There were no age-related differences for pointing measurements [Figure 5: Kruskal-Wallis Chi square: χ2_(12, N = 159)_ = 18.866; *p* = 0.09]. The average proprioceptive pointing of the overall sample was not significantly different from zero [*t*_(158)_ = −1.458, *p* = 0.147].

#### Visuo-proprioceptive pointing

The Gaussian distribution and the homogeneity of the variances (Levene's test) allowed testing handedness using an ANOVA. No effect of handedness was found [ANOVA (factor: right- vs. left-handed): *F*_(1, 155)_ = 0.300, *p* = 0.585]. Difference of variance between age groups was investigated using Levene's test and a difference was found [*F*_(12;143)_ = 2,171; *p* = 0.016]. Therefore, a Kruskal-Wallis was performed to investigate age effects. There were no age-related differences for pointing measurements (Figure 6: Kruskal-Wallis Chi square: χ2_(12, N = 156)_ = 14.749; *p* = 0.255).

#### Correlations between visuospatial assessments

Correlations between variables were investigated using Spearman's rank correlation. Correlations were computed on the whole sample as well as for each age group. Level of significance was corrected for multiple comparisons using a Bonferroni correction. Results for the whole sample are displayed in Table 4.

**Table 4:** Details of Spearman correlation between visuospatial tests.

	**Spearman correlation**	**1**	**2**	**3**	**4**	**5**	**6**
1	Star cancellation: All-star omission (*n*)						
2	Ogden figure copy (score)	0.25^*^					
3	Reading: Word omission (n)	−0.02	−0.09				
4	Line bisection: Average error (%)	−0.15	0.03	0.02			
5	Proprioceptive pointing: Average error (°)	0.00	0.13	0.04	0.07		
6	Visuo-proprioceptive pointing: Average error (°)	0.06	−0.05	−0.11	−0.08	−0.01	

* *p_corrected_ < 0.05*.

On the whole sample, a significant correlation between the score of the Ogden figure copy test and the total number of omitted stars was observed (*r*_*s*_ = 0.256; *p*_*corrected*_ = 0.001). No other significant correlation was found in the whole sample (all *p*_*corrected*_ > 1). Results of Spearman correlations within each age groups did not show significant correlation (all *p*_*corrected*_ > 0.288).

## Discussion

The present study investigated the development of visuospatial attention in TD children and developed pediatric reference values for both ego- and allocentric visuospatial assessments. Different developmental trajectories were highlighted in tests assessing ego- and allocentric visuospatial attention: the line bisection test and the visuo-proprioceptive pointing—the 2 purely allocentric tests—did not show any age-related changes. On the other hand, star cancellation, Ogden figure copy and reading tests—either egocentric or ego- and allocentric—presented an age-related performance. For assessment tools testing both accuracy and time, a change was observed in both variables. A leftward visuospatial attention bias was observed for the line bisection test.

### Differential developmental trajectories in ego- and allocentric tests

The present results do not support our initial hypothesis of children developing first egocentric visuospatial abilities and subsequently allocentric visuospatial abilities. This might be interpreted as a dissociation between spatial cognition and visuospatial attention. In this perspective, egocentric visuo-spatial abilities might develop later since they may depend on self-body representation that is developing throughout childhood until 10 years old (Brownell et al., 2007; Cowie et al., 2016) while the body *per se* is changing, and the motor control as well as the central representation have to adapt. No age related differences were found in the visuo-proprioceptive pointing task. This is not in agreement with the study of Hay (Hay, 1978), the difference between the studies might be related either to subtle differences between the tasks proposed or the fact that we included more age groups increasing thus the number of comparisons and potentially decreasing the statistical power.

However, we cannot exclude that the attentional load required by the assessment tools may play a role in the difference observed. Star cancellation is typically considered as measure of sustained and selective attention (Mitrushina et al., 2005), requiring a larger attentional load than line bisection or visuo-proprioceptive pointing. This may interfere since attention function is known to develop with age until 10–11 years old (Klenberg et al., 2001; Klimkeit et al., 2004).

The difference in the developmental trajectories of ego- and allocentric tests may also be related to different neural substrates underlying the performance of different visuospatial attention tasks (Milner and McIntosh, 2005; Pisella et al., 2013) with egocentric neglect being related to the fronto-parieto-temporal network and allocentric neglect to the parieto-temporal-occipital network (Chechlacz et al., 2010). A difference in the visual stream may also explain different neural substrates underlying both types of visuospatial attention as egocentric neglect appears to be linked predominantly to the dorsal visual pathways while the allocentric neglect may be related to the ventral visual stream (Medina et al., 2009; Corbetta and Shulman, 2011). Egocentric and allocentric visuospatial attention being related to different neural substrates may explain the different rates of development in different visuospatial attention assessments (Loenneker et al., 2011; Pisella et al., 2013). Future functional brain mapping studies [functional magnetic resonance imaging (fMRI) or electroencephalography (EEG)] could clarify the location and development of brain areas involved while performing the visuospatial attention tasks described here.

### Developmental trajectories of accuracy

Age differences were highlighted in the development of star cancellation, Ogden figure copy and reading tests. This suggests a development of visuo-spatial attention assessments with an egocentric component that has been previously highlighted. In a teddy-bear cancellation task in children from 3 to 8 years old, Laurent-Vannier et al. (2006) showed that typically developing children presented more teddy bear omissions before 6 years old than after. This development toward a better performance at 6 years old is congruent with our data and closely matches the time results observed in our star cancellation results and the development of our scores at the Ogden figure copy. The development observed in the Ogden figure copy is in line with previous studies using the copy of a complex figure, the Rey-Osterrieth figure, where children at the age of 6 omit almost no elements of the figure and improve until the age of 9 (Waber and Holmes, 1985; Akshoomoff and Stiles, 1995). Finally in the present study, the development in reading omissions is observed later since the test *per se* is not proposed under the age of 7.

Other factors than visuospatial attention could have influenced the changes observed in these results. The socio-economic status, for instance, may have had an influence. However, in the present study SEI was equally distributed among the different age groups and could therefore not explain the age effect observed. Some other non-visuospatial factors may have potentially influenced the results of the present study, such as the development of drawing and perception of object size (Waber and Holmes, 1985; Akshoomoff and Stiles, 1995; Bremner et al., 2000; Vinter and Marot, 2007; Lange-Küttner, 2008; Vinter et al., 2008). In the Ogden figure copy, our results closely match the development of some drawing abilities. Several authors reported a transition in drawing development around the age of 5–6 years old. Children around 5 years old start regulating the size of different objects drawn together (Lange-Küttner, 2008). Around the same age children change the way they plan their figure drawing, driven by the figure characteristics and no more by a left to right progression (Vinter et al., 2008). The difference observed in the Ogden figure copy between 5 and 6-years-old children could in part reflect the general development of drawing abilities in children and not only the development of visuospatial abilities. Possibly, more time in formal schooling influenced children's drawing abilities due to increased executional functioning and improved focus on cognitively demanding tasks (Brod et al., 2017). Though the development of drawing abilities may in part explain the changes observed in the Ogden Figure, they are not likely to influence the other egocentric assessments—i.e., not including drawing.

The star cancellation test may have been influenced by the ability of children to distinguish big stars from small stars. Previous studies reported that children at the age of five understand the concept of big and small and are able to distinguish a big object from a small one while the objects are simultaneously presented (Smith et al., 1985; Gelman and Ebeling, 1989). Therefore, our results showing a development in the score of star cancellation are probably more related to visuo-spatial attention than to the concept of object size.

A correlation was observed between scores (accuracy) in the Ogden figure copy and the total number of omitted stars. This correlation can be explained by the concomitant age-related changes in both tests and by the fact they both assess a combination of ego- and allocentric visuospatial attention.

### Developmental trajectories of time taken to perform the tasks

This study showed that the time needed to perform the star cancellation, the Ogden figure copy and the reading test decreases until the age of 12 years old. These results highlight a dissociation between the development of the time needed to perform the tasks and the performance/accuracy in the different tasks. This seems crucial to take into account when using these tests as assessments tools in children with potential deficits of visuospatial attention. Such a dissociation between speed and accuracy while performing a complex motor task have been previously described by Reis et al. (2009) who described the development of the relationship between these two parameters as the key element for inducing a skilled motor learning. At least regarding the Figure copy, the task required by the children might be considered as a complex motor task. Though the accuracy is maximal quite early, an improvement in performance is still possible through the change in the time needed to perform the task. Concerning the time needed to carry out the Ogden figure copy, Lange-Küttner highlighted in a previous study (1998) that 6-years-old children are copying angular shape more efficiently than the 4-year-olds. In comparison to younger children, older children were faster when copying angular forms than round forms. The authors suggested that these results could partially be explained by the geometric perfection of round forms in older children in comparison to the ubiquitous round forms in the drawings of younger children. Indeed a development of the time taken to copy the figure can be observed graphically (Figure 2). The age where a stabilization in the time needed to complete the copy is observed in our data-12 years old -matches in Belgium the start of secondary school (second cycle). The larger amount of exposure to taking notes during classes at this age may represent a potential bias and may play a part in the reduction of time needed to carry out the tests between 11 and 12 years old.

### Developmental trajectories of spatial bias

The present data show a leftwards pointing bias in the line bisection test, regardless of age. This leftwards bias has been described previously in healthy adults when performing line bisection tasks and pointing tasks (Jewell and McCourt, 2000; Richard et al., 2004). The leftwards bias found in healthy adults may be related to the attentional dominance of the right posterior parietal lobe, which is a critical region for performing the line bisection task in the near space (Bjoertomt et al., 2002; Chechlacz et al., 2012). There is some controversy regarding the presence of leftwards visuospatial bias in children when using the line bisection test (Bowers and Heilman, 1980). Studies in young children have shown a bias in function of the hand side used for performing the line bisection task (Dobler et al., 2001; Failla et al., 2003; Hausmann et al., 2003). In the present study, children were instructed to use their dominant hand only. Differences were found for line bisection according to the handedness, which is congruent with previous results by Pulsipher et al. (2009) reporting both leftwards and rightwards biases for line bisection in TD children. These biases seem to develop after 4 months of age since Lange-Küttner and Crichton (1999) showed that young infants present a bias of attention towards the right side of their visual field, which is comparable to neglect in adult patients. However, this attention bias resolves itself during the 4th month of life with the apparition of reaching in which infants show preference for reaching objects in their left visual field (Lange-Küttner and Crichton, 1999). At the same age previous studies reported that children can direct their gaze, engage and disengage their attention as well as shift it (Colombo, 2001).

### Limitations

A recruitment bias cannot be excluded as it was impossible to distinguish between parents whose children presented an exclusion criterion and parents who didn't agree to let their child participate. Also, the present reference values were collected in a school-aged pediatric population in Belgium and may differ according to ethnical and/or cultural origins.

Assessments were carried out in a fixed order. This procedure may have induced a bias due to fatigue. However, in the present study, the total time of testing was short and each task separately did not last more than 2 min. The visuospatial attention assessments were presented as games to the children. Though it seems unlikely that the fixed order of assessments may have induced fatigue, this possibility cannot be ruled out. Concerning the results of the reading text, as the text was written in lower case, younger children may have encountered more difficulties to read it. The measure of time could have been biased by some children trying to do the tests as fast as possible. Children were not asked to hurry during the tests, however, they knew that the time was being recorded which might be understood by some as a signal to perform the test as fast as possible.

## Conclusion

The present study describes pediatric reference values for visuospatial attention assessments and the development of visuospatial attention with age. Reference values are useful to detect visuospatial attention deficits in function of age and to describe pathological results. The use of visuospatial attention tests that are commonly applied in adults allows for an easy follow-up of visuospatial attention deficits during the transition from childhood to adulthood. Differential effects of age were observed with regards to the developmental trajectories of the different visuospatial assessments. Different neural substrates underlying different types of visuospatial attention (egocentric vs. allocentric) may explain differences of developmental speeds between visuospatial attention assessments.

## Ethics statement

This study was carried out in accordance with the recommendations of the ethics' committee of the Université catholique de Louvain with written informed consent from all subjects. All subjects gave written informed consent in accordance with the Declaration of Helsinki. The protocol was approved by the ethics' committee of the Université catholique de Louvain (Belgian ethics file number: B403201316810). Parents (or legal tutors) and children gave their written informed consent after receiving all information regarding the research protocol.

## Author contributions

YB, GI, and SH conceived the study. GI collected and analyzed the data and all the authors participated in the manuscript redaction. All the authors had complete access to the study data that support the publication and approved the manuscript.

### Conflict of interest statement

The authors declare that the research was conducted in the absence of any commercial or financial relationships that could be construed as a potential conflict of interest.
