# Supplementary material for: Development of Visuospatial Attention in Typically Developing Children
Source: Front Psychol. 2017 Dec 6;8:2064. doi: 10.3389/fpsyg.2017.02064 (PMC5724151; doi:10.3389/fpsyg.2017.02064)
Supplement: Supplementary file 1 [file Table1.PDF]

Appendix 1:

Testing protocol: visuospatial assessments

This testing protocol includes 6 different tests: 4 paper and pencil tests and 2 pointing tests:

Paper and pencil tests:

- Text reading test
- Ogden figure copy test
- Line bisection test
- Star cancellation test

Pointing tests:

- Proprioceptive pointing test
- Visuo-proprioceptive pointing test

Text reading:

Children have to read one full page of text aloud (appendix2).

Procedure:

The child is seated in front of a table adapted to his/her height. The sheet of paper with the text is presented on the table and aligned with the child's body midline (the line corresponding to the mid-sagittal plane of his/her body). While the instructions are given to the child, the text is hidden (i.e. the sheet of paper is turned face-down).

Instructions:

"There is a text on the back of this page. I will ask you to read this text out aloud as soon as I flip over the sheet of paper. Ready? Go!" (chrono)

To record by the experimenter on another copy sheet of the text:

- Time taken
- Indicate the omitted words

- Indicate the substitutions

Pay attention to:

- Hiding the text from the child during instructions
- Sheet midline aligned with the body midline of the child

Ogden figure copy:

This test consists of an A4 paper sheet with a drawing that children have to copy.

Procedure:

The child is seated in front of a table adapted to his/her height. The page with the drawing is placed in front of the child and aligned with his/her body midline. The experimenter gives the instructions to the child and then tells him/her to begin. The experimenter notes the time taken by the child. When the child has finished, he/she puts his/her pencil down on the table.

Instructions:

"On this page there is a drawing. I will ask you to reproduce it with the different details. It is not a race or an art contest. When you are sure to have copied everything, put your pencil down besides the page so I know that you have finished.

To record:

- Time taken

Pay attention to:

- Sheet midline aligned with the body midline of the child
- Do not give any clues to the child
    - Do not tell him/her if he/she forgot something
    - Answer neutrally if he/she asks if he/she copied everything ("I do not know, when you think you copied everything, put your pencil down beside the page")

Line bisection:

This test consists of three pages, presented one at a time. Each page consists of 10 lines that children have to bisect in two parts of equal length, without using anything but their eyes to assess the lines.

Procedure:

The child is seated in front of a table adapted to his/her height; the pages with the lines to bisect are presented in front of him/her, aligned with his/her body midline. The experimenter gives the instructions to the child, tells him/her to begin and starts to record the time at this moment (one time record for each page). The child bisects the different lines and puts his/her pencil down when he/she has finished. If the child forgot to bisect some lines, the experimenter tells him/her. If the child attempts to correct something, the experimenter tells him/her that it is against the instructions ("You cannot make any correction") and circles the first response, which will be the one used.

Instructions:

"In this test I will ask you to cut lines in two to show the midpoint of lines. Make a mark where you think the middle is. In the example, you can see a line. We asked a person to show the middle of the line by cutting the line in two and the person thought "the middle for me is there" and then the person made a mark at this place. I will ask you to do the same. Begin when I tell you "go"". (chrono)

To record:

- Time taken

Pay attention to:

- Only one answer per line; if corrections are made, circle the first mark (the one that will be used for scoring the test)
- Sheet midline aligned with the body midline of the child
- The child cannot use his/her fingers, pencil, etc., to assess the line lengths.
- The sheet has to remain in landscape position, the child can neither move nor rotate it.

Star cancellation:

This test consists of a full page presenting small and large stars as well as distractor words and letters. In this test, children have to cross out all the small stars presented on the page.

Procedure:

The child is seated in front of a table adapted to his/her height, the page with the test is presented in front of him/her, aligned with his/her body's midline. The experimenter gives the instructions to the child and records the time needed to complete the test. The test ends when the child puts his/her pencil down.

Instructions:

"In this test you can see small and large stars. I ask you to search all over the page for the small stars and to cross them all out. Like that (make the gesture of crossing a central small star). Once you think you have crossed all the small stars, I ask you to put your pencil down, so I will know that you have finished." (chrono)

To record:

- Time taken

Pay attention to:

- Sheet midline aligned with the child's body midline
- Sheet in landscape position
- Do not give any clues about the position of the stars:
  - If the child asks if he/she has crossed out all the small stars, give him/her a neutral answer ("if you think that you have crossed out all the small stars, it is ok. You have to check for yourself if you have crossed them out all.")
- Do not accept newly crossed stars after the child put his/her pencil down

Proprioceptive pointing:

In this test, children are asked to make free pointing movements straight ahead, in alignment with what they perceive as their body midline, while having their eyes closed. In order to measure the deviation between their pointing and their body midline, a page with the deviation expressed in degrees is used.

Procedure:

The child is seated in front of a table adapted to his/her height. His/her body midline is aligned with the 0° of the page used to record the deviation of his/her pointings. The child is asked to align his/her belly button with the 0° of the page and to straighten him/herself. The experimenter checks the child's position. Then, the experimenter explains the task to the child. The experimenter will also show to the child the procedure between the trials: the experimenter will put his/her own hand on the page, at a possible final position, and will put it back in the starting position (0°, edge of the page) while explaining to the child the he/she have to keep his/her eyes closed during the whole test and that the experimenter will take his/her finger and put it back between the different trials. Then, the child closes his/her eyes, points in front of him/her, the experimenter puts the child's index finger back in the starting position and the child points again for a total of 4 times.

Instructions:

"In this test, I will ask you to point straight ahead in front of you. This page will help me to measure the deviation of your pointings. I will ask you to align the midline of your body with the middle of the page so I can have a reliable measure of your pointing deviation. The middle of your body is your belly button and the middle of the page is there (the experimenter shows the middle of the page to the child). Align yourself by moving on your chair and/or by moving your chair (the child does not have to bend him/herself; then, the experimenter checks the alignment, corrects it if necessary and checks the distance between the child and the table). Here, straighten yourself, do not bend. I will ask you to point in front of you (the experimenter uses his/her hand to show the gesture) with your eyes closed. Once you have your eyes closed, you cannot reopen them until I tell you so. Thus, your hand will be on the page as mine is now (experimenter uses one of his/her hand with his/her index at the end of the page) and I will take it and put it back in the starting position and then you will do a new pointing (experimenter takes the finger of his/her hand posed on the page with his/her other hand and puts his/her hand back at the beginning of the page to show the procedure to the child). You cannot open your eyes between the trials. Ready? Go."

To record:

- Deviation in degrees of each trial: negative value for a deviation on the left side of the child and positive value for a deviation on the right side of the child

Pay attention to:

- Sign of the deviation, the experimenter often is in front of the child to take the measurements. A value is negative if pointing is on the left side of the target from the point of view of the child
- Alignment of the body with the midline of the sheet
- Check that the child’s eyes are closed during the whole procedure to avoid intermediary corrections

Visuo-proprioceptive pointing:

In this test, children are asked to point towards a visual target presented, above a wooden box, either in front of them (0°), on their left side (-21°) or on their right side (+21°) while having their arm hidden from eye sight. Each target is presented three times in a randomized way.

Procedure:

The child is seated in front of a table adapted to his/her height. A wooden box is used to record the deviation between the child’s pointing and the position of the different targets. The body midline of the child is aligned with the center of the box. The arm of the child is hidden by the box during the task. His/her hand is resting near the edge of the box. The experimenter presents the different targets to the child and asks him/her to point towards it (by touching the closed wall in Plexiglas of the box) and to leave his/her finger stable there where it hits the wall of the box. Once the child has touched the wall of the box, he/she cannot make any correction! The experimenter reports the different deviations with negative values if the child points to the left of the target and with positive values if the child points to the right of the target. The experimenter presents each target three times in a random order.

Instructions:

“In this test, you will have to point towards the pencil, that I will show you above the box. When you do it, you will not be able to see your arm. For this test, you have to align your body midline with the midline of the box. The middle of your body is your belly button and the middle of the box is here. Make sure to align the two by moving on your chair or by moving your chair. Here, now that you are correctly aligned, we can begin. Put your hand there and you will have to place it back in the same position between the different trials. Once you have touched the wall of the box to show me where the pencil is, you cannot make any correction and you have to leave your finger there until I tell you to go back to the starting position. Do you understand? Ok, let‘s start.”

To record:

- Deviation in degrees of the different pointings, negatives values for a deviation to the left and positive values for a deviation to the right of the targets.

Pay attention to:

- The child’s body midline alignment with the middle of the box.
- Corrections once the child has touched the wall of the box.
- Sign of the deviations.
