# Supplementary material for: Development of Visuospatial Attention in Typically Developing Children
Source: Front Psychol. 2017 Dec 6;8:2064. doi: 10.3389/fpsyg.2017.02064 (PMC5724151; doi:10.3389/fpsyg.2017.02064)
Supplement: Supplementary file 2 [file Table2.docx]

Star cancellation: All stars omission (n)

| Raw-score(n)/age Percentile | 5 yrs | 6 yrs | 7 yrs | 8 yrs | 9 yrs | 10 yrs | 11 yrs | 12 yrs | 13 yrs | 14 yrs | 15 yrs | 16 yrs | 17 yrs |
| --- | --- | --- | --- | --- | --- | --- | --- | --- | --- | --- | --- | --- | --- |
|  |  |  |  |  |  |  |  |  |  |  |  |  |  |
| Perc < 25 | 0,5 | 0 | 0 | 0,25 | 0 | 0 | 0 | 0 | 0 | 0 | 0 | 0 | 0 |
| Perc 25-50 | 0,5 - 3 | 0 - 1 | 0 - 1 | 0,25 - 2 | 0 - 1 | 0 - 1 | 0 - 0 | 0 - 0 | 0 - 0 | 0 - 0 | 0 - 0 | 0 - 0 | 0 - 0 |
| Perc 50-75 | 3 - 5 | 1 - 4 | 1 - 2,5 | 2 - 4 | 1 - 2,25 | 1 - 2 | 0 - 0 | 0 - 1 | 0 - 1 | 0 - 0 | 0 - 0 | 0 - 0 | 0 - 0 |
| Perc 75-95 | 5 - 9 | 4 - 8 | 2,5 - 9 | 4 - 9 | 2,25 - 4 | 2 - 4 | 0 - 1 | 1 - 1 | 1 - 3 | 0 - 2 | 0 - 0 | 0 - 0 | 0 - 4 |
| Perc >95 | > 9 | > 8 | > 9 | > 9 | > 4 | > 4 | > 1 | > 1 | > 3 | > 2 | > 0 | > 0 | > 4 |

Ogden figure copy: score (Range: 0-4)

| Raw-score/age Percentile | 5 yrs | 6 yrs | 7 yrs | 8 yrs | 9 yrs | 10 yrs | 11 yrs | 12 yrs | 13 yrs | 14 yrs | 15 yrs | 16 yrs | 17 yrs |
| --- | --- | --- | --- | --- | --- | --- | --- | --- | --- | --- | --- | --- | --- |
|  |  |  |  |  |  |  |  |  |  |  |  |  |  |
| Perc < 25 | 0 | 0 | 0 | 0 | 0 | 0 | 0 | 0 | 0 | 0 | 0 | 0 | 0 |
| Perc 25-50 | 0 - 1 | 0 - 0 | 0 - 0 | 0 - 0 | 0 - 0 | 0 - 0 | 0 - 0 | 0 - 0 | 0 - 0 | 0 - 0 | 0 - 0 | 0 - 0 | 0 - 0 |
| Perc 50-75 | 1 - 2 | 0 - 0 | 0 - 0,25 | 0 - 0,75 | 0 - 0 | 0 - 0 | 0 - 0 | 0 - 0 | 0 - 0 | 0 - 0 | 0 - 0 | 0 - 0 | 0 - 0 |
| Perc 75-95 | 2 - 2 | 0 - 2 | 0,25 - 1 | 0,75 - 1 | 0 - 0 | 0 - 0 | 0 - 0 | 0 - 0 | 0 - 1 | 0 - 0 | 0 - 0 | 0 - 0 | 0 - 0 |
| Perc >95 | > 2 | > 2 | > 1 | > 1 | > 0 | > 0 | > 0 | > 0 | > 1 | > 0 | > 0 | > 0 | > 0 |

| Raw-score(n)/age Percentile | 7 yrs | 8 yrs | 9 yrs | 10 yrs | 11 yrs | 12 yrs | 13 yrs | 14 yrs | 15 yrs | 16 yrs | 17 yrs |
| --- | --- | --- | --- | --- | --- | --- | --- | --- | --- | --- | --- |
|  |  |  |  |  |  |  |  |  |  |  |  |
| Perc < 25 | 0 | 0 | 0 | 0 | 0 | 0 | 0 | 0 | 0 | 0 | 0 |
| Perc 25-50 | 0 – 0 | 0 - 0 | 0 - 0 | 0 - 0 | 0 - 0 | 0 - 0 | 0 - 0 | 0 - 0 | 0 - 0 | 0 - 0 | 0 - 0 |
| Perc 50-75 | 0 – 0 | 0 - 0 | 0 - 0 | 0 - 1 | 0 - 1 | 0 - 1 | 0 - 0 | 0 - 1 | 0 - 0 | 0 - 1 | 0 - 0 |
| Perc 75-95 | 0 – 0 | 0 - 1 | 0 - 1 | 1 - 1 | 1 - 1 | 1 - 1 | 0 - 1 | 1 - 1 | 0 - 1 | 1 - 1 | 0 - 1 |
| Perc >95 | > 0 | > 1 | > 1 | > 1 | > 1 | > 1 | > 1 | > 1 | > 1 | > 1 | > 1 |

Reading omission: Word omission (n)

| Raw-score(%)/age Z-score | 5 yrs | 6 yrs | 7 yrs | 8 yrs | 9 yrs | 10 yrs | 11 yrs | 12 yrs | 13 yrs | 14 yrs | 15 yrs | 16 yrs | 17 yrs |
| --- | --- | --- | --- | --- | --- | --- | --- | --- | --- | --- | --- | --- | --- |
|  |  |  |  |  |  |  |  |  |  |  |  |  |  |
| <-3 | > -7.99 | > -17.3 | > -17.48 | > -12.95 | > -8.93 | > -10.57 | > -11.94 | > -8.81 | > -6.88 | > -9.16 | > -5.72 | > -7.08 | > -8.62 |
| -3;-2 | -7.99 ; -4.67 | -17.3 ; -12.29 | -17.48 ; -11.45 | -12.95 ; -8.89 | -8.93 ; -6.5 | -10.57 ; -7.67 | -11.94 ; -8.67 | -8.81 ; -5.72 | -6.88 ; -4.36 | -9.16 ; -6.33 | -5.72 ; -3.71 | -7.08 ; -4.93 | -8.62 ; -5.67 |
| -2;-1 | -4.67 ; -1.35 | -12.29 ; -7.28 | -11.45 ; -5.42 | -8.89 ; -4.83 | -6.5 ; -4.07 | -7.67 ; -4.77 | -8.67 ; -5.4 | -5.72 ; -2.63 | -4.36 ; -1.84 | -6.33 ; -3.5 | -3.71 ; -1.7 | -4.93 ; -2.78 | -5.67 ; -2.72 |
| -1;0 | -1.35 ; 1.97 | -7.28 ; -2.27 | -5.42 ; 0.61 | -4.83 ; -0.77 | -4.07 ; -1.64 | -4.77 ; -1.87 | -5.4 ; -2.13 | -2.63 ; 0.46 | -1.84 ; 0.68 | -3.5 ; -0.67 | -1.7 ; 0.31 | -2.78 ; -0.63 | -2.72 ; 0.23 |
| 0;1 | 1.97 ; 5.29 | -2.27 ; 2.74 | 0.61 ; 6.64 | -0.77 ; 3.29 | -1.64 ; 0.79 | -1.87 ; 1.03 | -2.13 ; 1.14 | 0.46 ; 3.55 | 0.68 ; 3.2 | -0.67 ; 2.16 | 0.31 ; 2.32 | -0.63 ; 1.52 | 0.23 ; 3.18 |
| 1;2 | 5.29 ; 8.61 | 2.74 ; 7.75 | 6.64 ; 12.67 | 3.29 ; 7.35 | 0.79 ; 3.22 | 1.03 ; 3.93 | 1.14 ; 4.41 | 3.55 ; 6.64 | 3.2 ; 5.72 | 2.16 ; 4.99 | 2.32 ; 4.33 | 1.52 ; 3.67 | 3.18 ; 6.13 |
| 2;3 | 8.61 ; 11.93 | 7.75 ; 12.76 | 12.67 ; 18.7 | 7.35 ; 11.41 | 3.22 ; 5.65 | 3.93 ; 6.83 | 4.41 ; 7.68 | 6.64 ; 9.73 | 5.72 ; 8.24 | 4.99 ; 7.82 | 4.33 ; 6.34 | 3.67 ; 5.82 | 6.13 ; 9.08 |
| 3 | 11.93 | 12.76 | 18.7 | 11.41 | 5.65 | 6.83 | 7.68 | 9.73 | 8.24 | 7.82 | 6.34 | 5.82 | 9.08 |

Line bisection test: Average error (%)

| Raw-score(°)/age Z-score | 5 yrs | 6 yrs | 7 yrs | 8 yrs | 9 yrs | 10 yrs | 11 yrs | 12 yrs | 13 yrs | 14 yrs | 15 yrs | 16 yrs | 17 yrs |
| --- | --- | --- | --- | --- | --- | --- | --- | --- | --- | --- | --- | --- | --- |
|  |  |  |  |  |  |  |  |  |  |  |  |  |  |
| <-3 | > -23.3 | > -14.99 | > -14.03 | > -16.9 | > -10.72 | > -14.62 | > -11.9 | > -11.11 | > -9.11 | > -10.29 | > -6.46 | > -11.8 | > -11.85 |
| -3;-2 | -23.3 ; -14.34 | -14.99 ; -11.02 | -14.03 ; -10.12 | -16.9 ; -12.12 | -10.72 ; -7.56 | -14.62 ; -10.69 | -11.9 ; -9.02 | -11.11 ; -7.44 | -9.11 ; -6.37 | -10.29 ; -7.13 | -6.46 ; -4.24 | -11.8 ; -7.92 | -11.85 ; -8.63 |
| -2;-1 | -14.34 ; -5.38 | -11.02 ; -7.05 | -10.12 ; -6.21 | -12.12 ; -7.34 | -7.56 ; -4.4 | -10.69 ; -6.76 | -9.02 ; -6.14 | -7.44 ; -3.77 | -6.37 ; -3.63 | -7.13 ; -3.97 | -4.24 ; -2.02 | -7.92 ; -4.04 | -8.63 ; -5.41 |
| -1;0 | -5.38 ; 3.58 | -7.05 ; -3.08 | -6.21 ; -2.3 | -7.34 ; -2.56 | -4.4 ; -1.24 | -6.76 ; -2.83 | -6.14 ; -3.26 | -3.77 ; -0.1 | -3.63 ; -0.89 | -3.97 ; -0.81 | -2.02 ; 0.2 | -4.04 ; -0.16 | -5.41 ; -2.19 |
| 0;1 | 3.58 ; 12.54 | -3.08 ; 0.89 | -2.3 ; 1.61 | -2.56 ; 2.22 | -1.24 ; 1.92 | -2.83 ; 1.1 | -3.26 ; -0.38 | -0.1 ; 3.57 | -0.89 ; 1.85 | -0.81 ; 2.35 | 0.2 ; 2.42 | -0.16 ; 3.72 | -2.19 ; 1.03 |
| 1;2 | 12.54 ; 21.5 | 0.89 ; 4.86 | 1.61 ; 5.52 | 2.22 ; 7 | 1.92 ; 5.08 | 1.1 ; 5.03 | -0.38 ; 2.5 | 3.57 ; 7.24 | 1.85 ; 4.59 | 2.35 ; 5.51 | 2.42 ; 4.64 | 3.72 ; 7.6 | 1.03 ; 4.25 |
| 2;3 | 21.5 ; 30.46 | 4.86 ; 8.83 | 5.52 ; 9.43 | 7 ; 11.78 | 5.08 ; 8.24 | 5.03 ; 8.96 | 2.5 ; 5.38 | 7.24 ; 10.91 | 4.59 ; 7.33 | 5.51 ; 8.67 | 4.64 ; 6.86 | 7.6 ; 11.48 | 4.25 ; 7.47 |
| 3 | 30.46 | 8.83 | 9.43 | 11.78 | 8.24 | 8.96 | 5.38 | 10.91 | 7.33 | 8.67 | 6.86 | 11.48 | 7.47 |

Proprioceptive pointing: Average error (°)

| Raw-score(°)/age Z-score | 5 yrs | 6 yrs | 7 yrs | 8 yrs | 9 yrs | 10 yrs | 11 yrs | 12 yrs | 13 yrs | 14 yrs | 15 yrs | 16 yrs | 17 yrs |
| --- | --- | --- | --- | --- | --- | --- | --- | --- | --- | --- | --- | --- | --- |
|  |  |  |  |  |  |  |  |  |  |  |  |  |  |
| <-3 | > -6.49 | > -3.6 | > -3.74 | > -2.65 | > -1.82 | > -2.76 | > -2.03 | > -3.04 | > -2.3 | > -2.55 | > -1.45 | > -2.04 | > -1.99 |
| -3;-2 | -6.49 ; -4.27 | -3.6 ; -2.25 | -3.74 ; -2.5 | -2.65 ; -1.49 | -1.82 ; -1.08 | -2.76 ; -1.89 | -2.03 ; -1.28 | -3.04 ; -1.93 | -2.3 ; -1.38 | -2.55 ; -1.68 | -1.45 ; -0.96 | -2.04 ; -1.44 | -1.99 ; -1.29 |
| -2;-1 | -4.27 ; -2.05 | -2.25 ; -0.9 | -2.5 ; -1.26 | -1.49 ; -0.33 | -1.08 ; -0.34 | -1.89 ; -1.02 | -1.28 ; -0.53 | -1.93 ; -0.82 | -1.38 ; -0.46 | -1.68 ; -0.81 | -0.96 ; -0.47 | -1.44 ; -0.84 | -1.29 ; -0.59 |
| -1;0 | -2.05 ; 0.17 | -0.9 ; 0.45 | -1.26 ; -0.02 | -0.33 ; 0.83 | -0.34 ; 0.4 | -1.02 ; -0.15 | -0.53 ; 0.22 | -0.82 ; 0.29 | -0.46 ; 0.46 | -0.81 ; 0.06 | -0.47 ; 0.02 | -0.84 ; -0.24 | -0.59 ; 0.11 |
| 0;1 | 0.17 ; 2.39 | 0.45 ; 1.8 | -0.02 ; 1.22 | 0.83 ; 1.99 | 0.4 ; 1.14 | -0.15 ; 0.72 | 0.22 ; 0.97 | 0.29 ; 1.4 | 0.46 ; 1.38 | 0.06 ; 0.93 | 0.02 ; 0.51 | -0.24 ; 0.36 | 0.11 ; 0.81 |
| 1;2 | 2.39 ; 4.61 | 1.8 ; 3.15 | 1.22 ; 2.46 | 1.99 ; 3.15 | 1.14 ; 1.88 | 0.72 ; 1.59 | 0.97 ; 1.72 | 1.4 ; 2.51 | 1.38 ; 2.3 | 0.93 ; 1.8 | 0.51 ; 1 | 0.36 ; 0.96 | 0.81 ; 1.51 |
| 2;3 | 4.61 ; 6.83 | 3.15 ; 4.5 | 2.46 ; 3.7 | 3.15 ; 4.31 | 1.88 ; 2.62 | 1.59 ; 2.46 | 1.72 ; 2.47 | 2.51 ; 3.62 | 2.3 ; 3.22 | 1.8 ; 2.67 | 1 ; 1.49 | 0.96 ; 1.56 | 1.51 ; 2.21 |
| 3 | 6.83 | 4.5 | 3.7 | 4.31 | 2.62 | 2.46 | 2.47 | 3.62 | 3.22 | 2.67 | 1.49 | 1.56 | 2.21 |

Visuo-proprioceptive pointing: Average error (°)
