# Supplementary material for: Development of Visuospatial Attention in Typically Developing Children
Source: Front Psychol. 2017 Dec 6;8:2064. doi: 10.3389/fpsyg.2017.02064 (PMC5724151; doi:10.3389/fpsyg.2017.02064)
Supplement: Supplementary file 3 [file Table3.DOCX]

| Test | Variable | Age class (yrs) | Subjects (n=) | Mean | Standard Deviation | 95%-confidence interval |
| --- | --- | --- | --- | --- | --- | --- |
|  |  |  |  |  |  |  |
| Line bisection: line of 5cm and less | average error (%) | 5 | 8 | 4,74 | 7,486 | -10,23 - 19,71 |
|  |  | 6 | 11 | -1,66 | 10,937 | -23,54 - 20,21 |
|  |  | 7 | 13 | -5,92 | 4,233 | -14,39 - 2,55 |
|  |  | 8 | 12 | -3,85 | 4,992 | -13,83 - 6,14 |
|  |  | 9 | 13 | -3,31 | 3,731 | -10,78 - 4,15 |
|  |  | 10 | 13 | -5,23 | 4,064 | -13,36 - 2,9 |
|  |  | 11 | 8 | -3,82 | 2,542 | -8,91 - 1,26 |
|  |  | 12 | 13 | -1,89 | 4,724 | -11,34 - 7,56 |
|  |  | 13 | 17 | -2,68 | 3,948 | -10,58 - 5,21 |
|  |  | 14 | 13 | -2,40 | 3,519 | -9,44 - 4,64 |
|  |  | 15 | 12 | -1,04 | 3,585 | -8,21 - 6,13 |
|  |  | 16 | 12 | -0,97 | 3,343 | -7,65 - 5,72 |
|  |  | 17+ | 11 | -2,51 | 3,772 | -10,06 - 5,03 |
| Line bisection: line of 10cm and less | average error (%) | 5 | 8 | 6,01 | 12,610 | -19,21 - 31,23 |
|  |  | 6 | 11 | -0,91 | 8,312 | -17,53 - 15,72 |
|  |  | 7 | 13 | -1,99 | 4,704 | -11,4 - 7,42 |
|  |  | 8 | 12 | -0,10 | 6,017 | -12,14 - 11,93 |
|  |  | 9 | 13 | 0,79 | 3,332 | -5,87 - 7,45 |
|  |  | 10 | 13 | -0,65 | 4,995 | -10,64 - 9,34 |
|  |  | 11 | 8 | -1,94 | 4,219 | -10,38 - 6,5 |
|  |  | 12 | 13 | 1,65 | 3,580 | -5,51 - 8,81 |
|  |  | 13 | 17 | 0,49 | 4,490 | -8,49 - 9,47 |
|  |  | 14 | 13 | 0,69 | 4,487 | -8,28 - 9,66 |
|  |  | 15 | 12 | 2,18 | 3,301 | -4,43 - 8,78 |
|  |  | 16 | 12 | 2,01 | 5,215 | -8,42 - 12,44 |
|  |  | 17+ | 11 | -0,59 | 3,981 | -8,55 - 7,37 |
| Line bisection: line of 15cm and less | average error (%) | 5 | 8 | 1,16 | 12,802 | -24,44 - 26,77 |
|  |  | 6 | 11 | -5,98 | 9,414 | -24,8 - 12,85 |
|  |  | 7 | 13 | -1,17 | 6,701 | -14,57 - 12,24 |
|  |  | 8 | 12 | -3,47 | 5,553 | -14,58 - 7,63 |
|  |  | 9 | 13 | -1,36 | 4,757 | -10,88 - 8,15 |
|  |  | 10 | 13 | -3,49 | 4,872 | -13,23 - 6,26 |
|  |  | 11 | 8 | -3,76 | 2,903 | -9,56 - 2,05 |
|  |  | 12 | 13 | -0,25 | 3,532 | -7,31 - 6,82 |
|  |  | 13 | 17 | -0,25 | 2,225 | -4,7 - 4,2 |
|  |  | 14 | 13 | -1,62 | 3,051 | -7,72 - 4,48 |
|  |  | 15 | 12 | 0,03 | 3,040 | -6,05 - 6,11 |
|  |  | 16 | 12 | -1,43 | 5,080 | -11,59 - 8,73 |
|  |  | 17+ | 11 | -3,15 | 3,633 | -10,41 - 4,12 |
| Line bisection: line of more than 15cm | average error (%) | 5 | 8 | -6,61 | 10,945 | -28,5 - 15,28 |
|  |  | 6 | 11 | -6,11 | 8,459 | -23,02 - 10,81 |
|  |  | 7 | 13 | 2,01 | 9,108 | -16,21 - 20,23 |
|  |  | 8 | 12 | -3,84 | 9,691 | -23,23 - 15,54 |
|  |  | 9 | 13 | -1,08 | 5,510 | -12,1 - 9,93 |
|  |  | 10 | 13 | -0,88 | 4,565 | -10,01 - 8,25 |
|  |  | 11 | 8 | -1,75 | 7,611 | -16,97 - 13,47 |
|  |  | 12 | 13 | 0,20 | 5,749 | -11,3 - 11,7 |
|  |  | 13 | 17 | 0,53 | 4,008 | -7,49 - 8,55 |
|  |  | 14 | 13 | 1,28 | 4,382 | -7,48 - 10,05 |
|  |  | 15 | 12 | 0,61 | 4,669 | -8,73 - 9,95 |
|  |  | 16 | 12 | 0,81 | 5,575 | -10,34 - 11,96 |
|  |  | 17+ | 11 | -2,92 | 3,317 | -9,56 - 3,71 |

**Supplementary Table 3:** Pediatric reference values by age group for the different line lengths of the line bisection test.
