# Supplementary material for: Development of Visuospatial Attention in Typically Developing Children
Source: Front Psychol. 2017 Dec 6;8:2064. doi: 10.3389/fpsyg.2017.02064 (PMC5724151; doi:10.3389/fpsyg.2017.02064)
Supplement: Supplementary file 5 [file Image1.PDF]

Appendix 2

L'ARBRE QUI CHANTAIT

Il y a très, très longtemps, un vieux sorcier entreprit un long voyage.

Un jour qu'il avait tant et tant marché qu'il ne sentait plus ses pieds, il décida de chercher un endroit pour se reposer.

C'est alors qu'il entendit soudain chanter. Ce n'était pas un chant comme celui des oiseaux, ni comme celui du vent à travers les feuilles, mais une voix claire, qui prononçait des mots qu'il ne comprenait point.
