# Supplementary material for: Development of Visuospatial Attention in Typically Developing Children
Source: Front Psychol. 2017 Dec 6;8:2064. doi: 10.3389/fpsyg.2017.02064 (PMC5724151; doi:10.3389/fpsyg.2017.02064)

Appendix 6: Drawing evolution: absence of 3D was not scored as an error.

5 years old

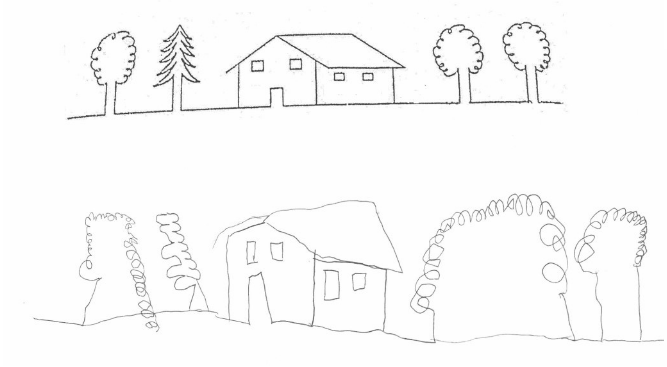

6 years old

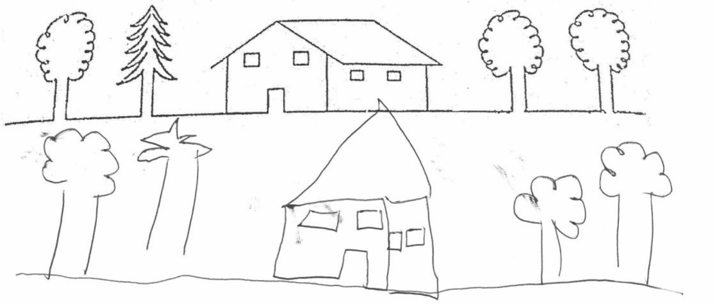

7 years old

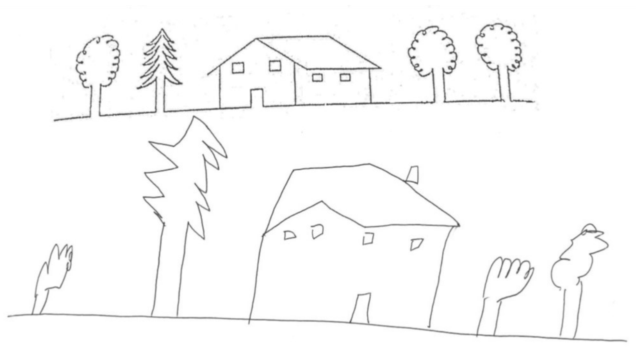

8 years old

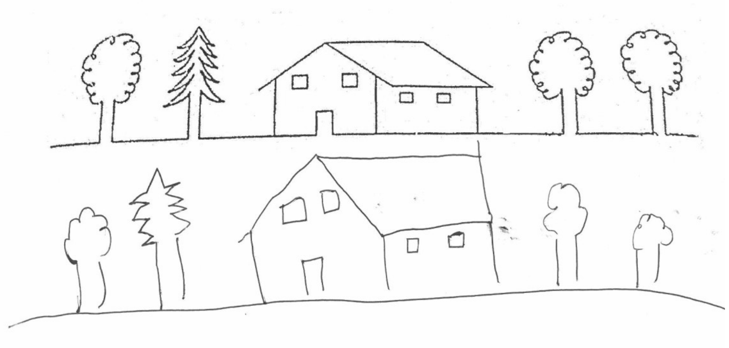

9 years old

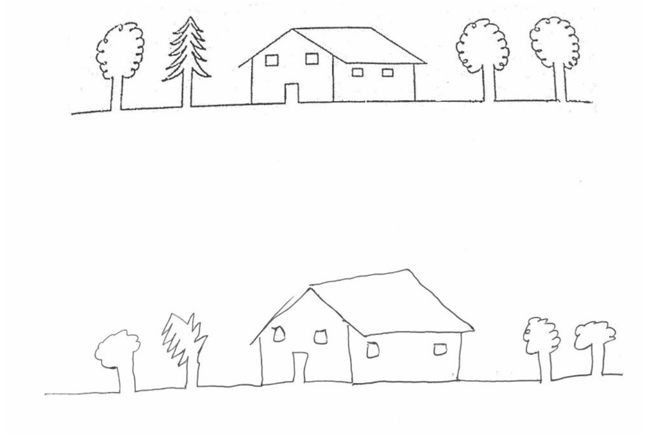

10 years old

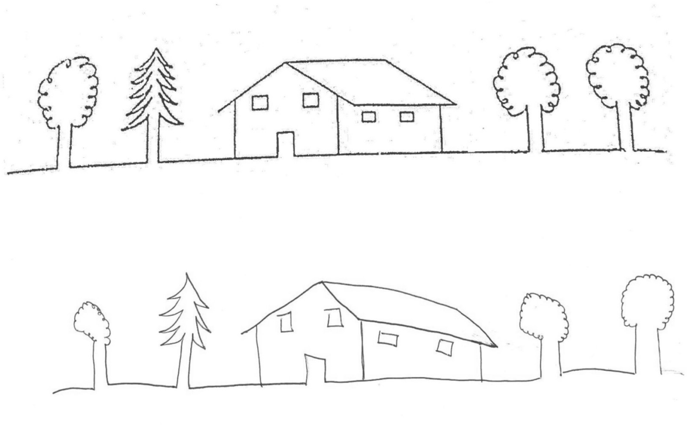

13 years old

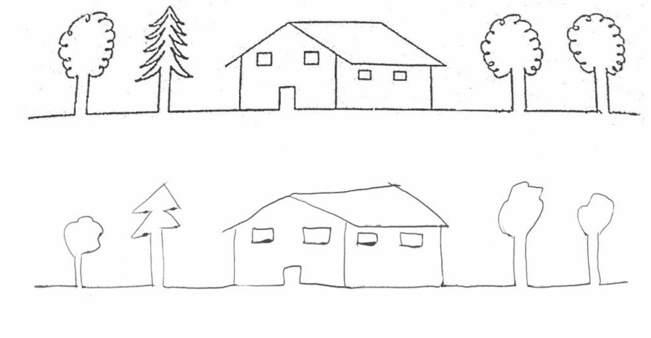

17 years old

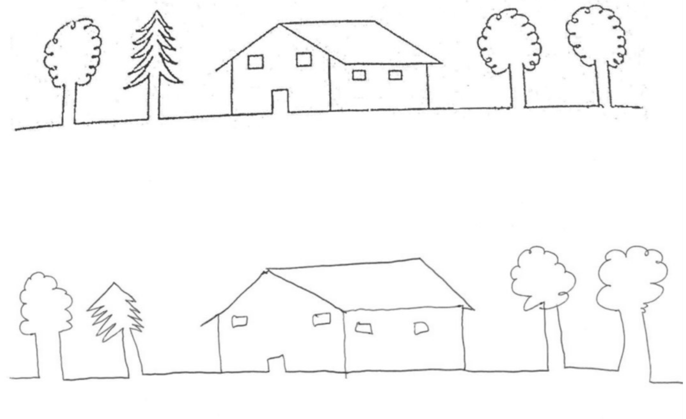

Supplement: Supplementary file 6 [file Image2.PDF]
